# Bronchodilator reversibility testing in morbidly obese non-smokers: fluticasone/salmeterol efficacy versus salbutamol bronchodilator

**DOI:** 10.1186/s12890-023-02682-3

**Published:** 2023-10-09

**Authors:** Mona Ibrahim Ahmed, Randa Ibrahim Ahmed, Hasnaa Osama, Amira Karam Khalifa, Abdullah Ali Alshehri, Gaber El-Saber Batiha, Walaa A Negm, Marwa Kamal

**Affiliations:** 1 Department of chest Ds & TB, Faculty of Medicine, Fayoum University, Fayoum, Egypt; 2 Clinical Pharmacy Department, Faculty of Pharmacy, Beni-suef University, Beni-suef, Egypt; 3 Department of Medical pharmacology, Kasr El-Ainy School of Medicine, Cairo University, El Manial, Cairo, 11562 Egypt; 4 Department of Medical Pharmacology, Nahda Faculty of Medicine, Beni Suef, 62521 Egypt; 5 Department of Clinical Pharmacy, College of Pharmacy, Taif University, Al Hawiyah, Taif, Saudi Arabia; 6 Department of Pharmacology and Therapeutics, Faculty of Veterinary Medicine, Damanhour University, Damanhour, AlBeheira, 22511 Egypt; 7 Department of Pharmacognosy, Faculty of Pharmacy, Tanta University, Tanta, 31527 Egypt; 8 Clinical Pharmacy Department, Faculty of Pharmacy, Fayoum University, Fayoum, 63514 Egypt

**Keywords:** Obese, fluticasone/salmeterol, Salbutamol, Bronchodilators, Spirometry, Reversibility test

## Abstract

A positive response in reversibility testing is widely used to diagnose patients with airway limitations. However, despite its simple procedure, it doesn’t accurately reflect the exact airway irreversibility. This study aimed to investigate the efficacy of a bronchodilation reversibility test using salbutamol and fluticasone/salmeterol combination in obese non-smoker subjects.

The study included patients without a history of obstructive lung disease or bronchodilators. A sub-classification of patients based on body mass index (BMI) was carried out into normal (< 24.9 kg/m^2^), overweight (25-29.9 kg/m^2^), and obese (BMI ≥ 30). Spirometry measurements were performed before and after salbutamol or fluticasone/salmeterol administration.

The study included 415 (49.9% male) patients with a mean age of 40.92 ± 10.86 years. Obese subjects showed a high prevalence of restrictive patterns (23.4%), with non-significantly lower spirometric values compared to normal and overweight subjects (p > 0.05). The magnitude of bronchodilation, as identified by spirometry, following fluticasone/salmeterol was higher in all participants, with a significant increase in obese subjects with a p-value of 0.013, 0.002, and 0.035 for FEV_1_, FEV_1_% predicted, and FEV_1_/FVC, respectively.

Fluticasone/salmeterol combination increases FEV_1_, FEV_1_% of predicted, and FEV_1_/FVC ratio than the conventional test using salbutamol inhaler, and it can be a potential candidate for assessment of airway obstruction using reversibility test, especially among the obese population.

## Introduction

Asthma and chronic obstructive pulmonary disease (COPD) are complex chronic diseases with a high prevalence across the globe. Asthma is an inflammatory disease characterised by airway hyperresponsiveness that is mostly reversible, while COPD is an irreversible, progressive airway obstruction [1]. A diagnosis of obstructive pulmonary disease usually depends on symptomatic features. Then airflow expiration tests are needed to confirm the suspected diagnosis, thereby influencing the pattern of medical care and drug preferences [2]. Spirometry is pivotal in assessing bronchodilator reversibility. It is considered the standard gold standard for diagnosing diseases with obstructive airways in general practice. Reversibility testing measures the airflow expiration response after inhaling a bronchodilator. A positive test is defined as an increase in FEV1 of more than 12% from baseline or a 200-ml increase, according to the American Thoracic Society (ATS). In comparison, the European Respiratory Society (ERS) recommended a change of more than 9% of the predicted FEV1 as a hallmark for asthma confirmation [1].

Simple bronchodilator reversibility testing is safe with relevantly few clinical risks and can be carried out promptly. However, its results have limited reproducibility and accuracy. Since it depends on several factors, such as the patient’s maximal effort and the bronchodilator used [3].

In addition, the defined cut-offs of the positive reversibility test may fail to detect those with low FEV1 at baseline or those with preserved lung function or large lung volumes [4].

Obesity is associated with a decline in lung function. It can also worsen the manifestations of airway obstruction and severity and decrease the response to conventional therapies among patients with asthma and COPD [5, 6]. Numerous investigations have documented the occurrence of misdiagnosis of obstructive airways in individuals with morbid obesity [7]. These studies have elucidated that the elevated body mass index (BMI) in this population may contribute to diagnostic ambiguity and the possibility of misclassifying asthma diagnoses. The impact of body mass index (BMI) on the efficacy of asthma drugs and conventional bronchodilators has been documented to have a detrimental effect, resulting in compromised asthma control within the obese population.

Therefore, combination medications may be required to achieve considerable airway response. We hypothesize that the obese population may show limited response to short-acting bronchodilators in early reversibility tests, contributing to the misdiagnosis of obstructive airway disease. Inevitably, an incorrect asthma diagnosis may cause inappropriate treatment, with a higher potential of side effects and cost burden [7]. Few studies investigated the efficacy of different bronchodilator combinations in early reversibility testing, such as LABAs/inhaled corticosteroids, levosalbutamol/ipratropium, or Glycopyrronium bromide and salbutamol combination [8–10]. However, none of these studies investigated the effect of these combinations in early reversibility tests amongst the obese population.

Hence, our study aimed to investigate the efficacy of fluticasone/salmeterol relative to salbutamol in early reversibility testing among a population with different body mass indexes.

## Materials and methods

### Study population

This observational study was conducted at Fayoum University Hospital, Fayoum, Egypt, from October 2021 to January 2022. Symptomatic patients with cough, dyspnea, and/or wheezing were screened at the first visit for recruitment. Eligibility criteria were: (a) age above 18 years old; (b) patients who had never received short- or long-acting bronchodilators before or within the past 12 h before the reversibility test. We excluded patients who were current smokers or ex-smokers. Oral corticosteroid users were also excluded. The study protocol was conducted according to ethical guidelines and approved by Fayoum University’s ethical committee. All subjects were instructed about the study procedure, and informed consent was obtained.

### Study design

Eligible participants were randomized to receive salbutamol (Ventolin®, two puffs of 200 µg/ two puffs; total dose 400 µg or fluticasone/ salmeterol (Seretide Evohaler®, 125/25µg/ two puffs; total dose 250/50µg dose) bronchodilators combination using a pressurized metered-dose inhaler (pMDI) with a spacer for both groups. Subgroup categorization of each group was performed according to body mass index (BMI) into normal weight (18.5–24.9 kg/m^2^), overweight (25-29.9 kg/m^2^) and obese (BMI ≥ 30). A comprehensive clinical assessment, including complete medical history recording, physical examination, and PFT, was performed for all the study participants.

Initially, participants underwent pre-bronchodilator spirometry using the Spirodoc S/N instrument (MIR Spirodoc, Spiro + Oxi, Roma, Italy) according to the American Thoracic Society (ATS) standards. The basal forced expiratory volume in one second (FEV_1_), forced vital capacity (FVC), and FEV_1_/FVC was measured in the seated position, and the best value of three successful maneuvers was recorded. Then, subjects were allocated to inhaled bronchodilators with the specified doses, and spirometry was repeated 15 min later. Reversibility was considered positive in patients whose FEV_1_ showed at least 12% improvement from basal value and at least ≥ 200 mL increase after bronchodilator administration [11, 12]. The normal and percent predicted values were estimated from the reference values of the Global Lung Initiative (GLI).

### Statistical analysis

Analyses were conducted with SPSS 16.0 (Inc., Chicago, IL). The changes in spirometric outcomes by each bronchodilator regimen were determined by the analysis of variance (ANOVA) test, and P-value < 0.05 was considered statistically significant. A student t-test was used to investigate the differences in demographic characteristics and per-bronchodilator spirometric outcomes in salbutamol and fluticasone/salmeterol groups. A Chi-square test was used to compare the proportion of patients achieving post-bronchodilator improvement in spirometric variables in the study groups. Quantitative data were expressed as mean ± SD, while categorical data were expressed as case frequencies (n) and percentages.

## Results

### Study sample characteristics

In total, 426 patients were initially screened. Only 415 subjects met the inclusion criteria and were willing to participate in the study. The difference in baseline characteristics of the study groups was statistically non-significant (p > 0.05). The distribution of demographic variables and baseline lung functions on initial spirometry are listed in Table 1. The recruited subjects’ ages ranged from 19 to 78 years, with a mean value of 40.92 ± 10.86 years. There were 207 males (49.9%) and 208 females (50.1%) participants in the study. The difference in body mass index between the normal, overweight, and obese was statistically significant (P < 0.001) with an average of 22.06 ± 2.2, 27.81 ± 1.37, and 35.67 ± 4.58, respectively, for the salbutamol group and 23.2 ± 5.83, 28.46 ± 1.8, and 36.2 ± 6.3, respectively for salmeterol/fluticasone group.

**Table 1:** Baseline demographic and lung functions on initial spirometry

Variables	Salbutamol group (*n* = 209)				Fluticasone/Salmeterol group (*n* = 206)				^1^P-value
	*Normal*	*overweight*	*Obese*	**P-value**	*Normal*	*overweight*	*obese*	**P-value**	
Number of participants (%)	70 (33.49)	69 (33.01)	70 (33.49)		68 (33.01)	68 (33.01)	70 (33.98)		
Age (years)	37.93 ± 12.4	38.49 ± 14.5	42.95 ± 13.8	0.059	39.14 ± 8.1	41.85 ± 9.3	45.14 ± 11.9	0.036	NS
Gender (M/F)	46/24	33/36	26/44	0.094	33/35	38/30	31/39	0.174	NS
BMI (kg/m^2^)	22.96 ± 2.21	27.81 ± 1.37	35.67 ± 4.58	< 0.001*	23.2 ± 5.83	28.06 ± 1.8	35.2 ± 6.3	< 0.001*	NS
FEV_1_ (L)	3.15 ± 1.2	2.93 ± 0.81	2.79 ± 1.03	0.115	3.72 ± 1.46	3.29 ± 1.28	3.24 ± 1.17	0.064	NS
FEV_1_% of predicted	76.2 ± 12.6	74.5 ± 16.4	72.41 ± 18.3	0.372	78.28 ± 12.5	77.05 ± 11.3	76.56 ± 12.9	0.699	NS
FVC (L)	3.45 ± 1.14	3.41 ± 0.78	3.34 ± 0.97	0.796	4.16 ± 1.41	3.92 ± 1.36	3.64 ± 1.27	0.079	NS
FVC% of predicted	89.78 ± 10.8	87.15 ± 12.37	86.63 ± 14.52	0.281	90.18 ± 12.8	87.22 ± 10.41	85.34 ± 13.62	0.068	NS
FEV_1_/FVC	73.88 ± 17.61	73.15 ± 19.28	70.94 ± 18.13	0.615	76.83 ± 15.8	74.52 ± 14.7	71.24 ± 16.51	0.112	NS

M/F: male/Female; FEV1: Forced expiratory volume in 1 s; FVC: Forced vital capacity

^1^The p-value difference between the salbutamol and Fluticasone/salmeterol groups

*Statistically significant differences within groups

### Pre-bronchodilator spirometry

Spirometric values of obese were lower when compared to normal and overweight subjects. However, the difference was non-statistically significant (p > 0.05). Also, restrictive abnormal pattern of lung function tests was highly prevalent among the obese participants (23.4%). Obstructive and mixed patterns frequencies were 2.14% and 1.43%, respectively, in obese subjects.

### Reversibility rest outcomes

**Fig. 1:**
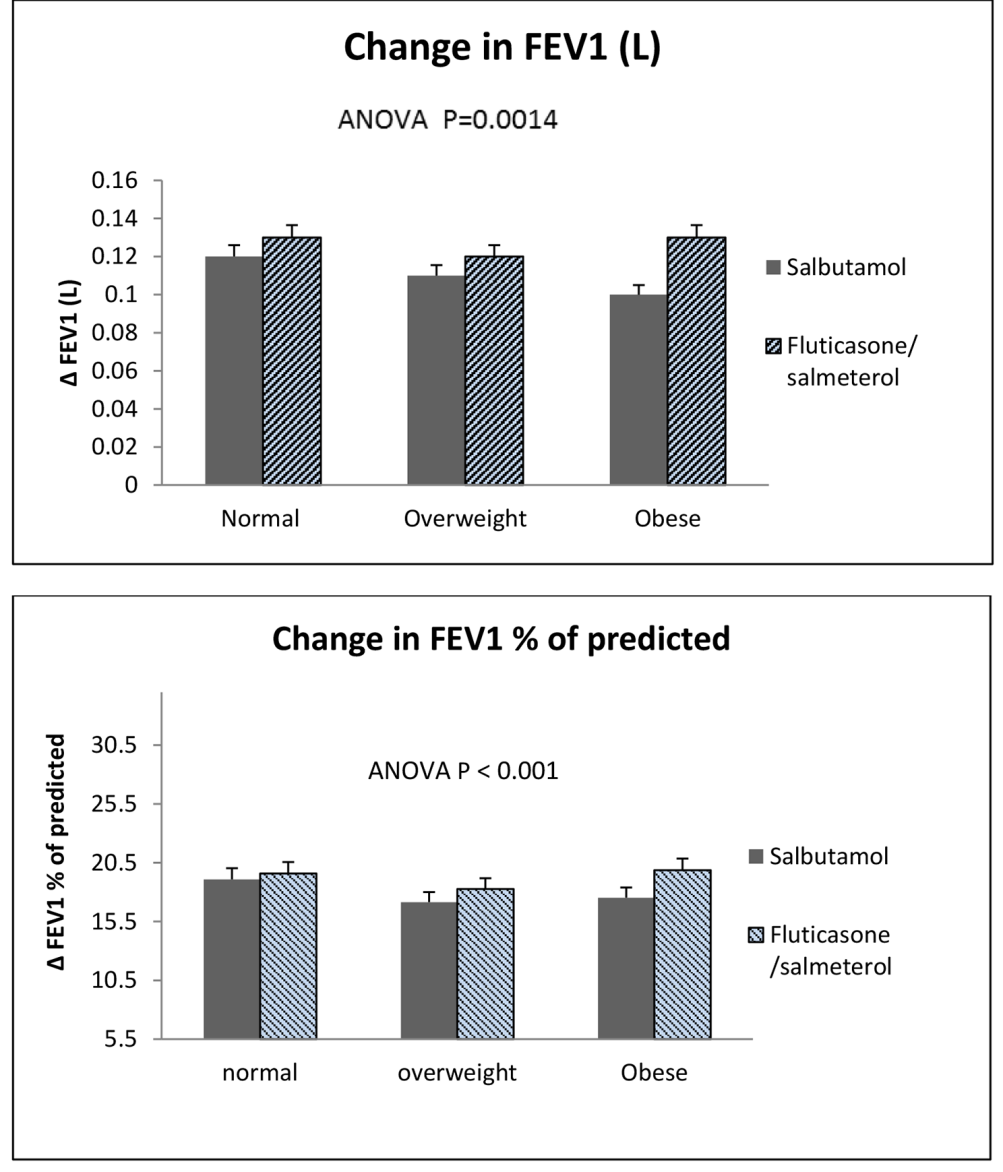
Mean change in spirometric results; **(a)** FEV_1_ (L) and **(b)** FEV_1_% of predicted (FEV_1_%) within the study groups

Following baseline spirometry, the reversibility test was performed using salbutamol and salmeterol/fluticasone combination in two randomized groups with subgroup categorization into normal, overweight, and obese. Comparisons between post-bronchodilator spirometric results in salbutamol versus fluticasone/salmeterol combination are illustrated in Table 2. The values of FEV_1_ and FEV_1_/FVC were higher in the salmeterol/fluticasone group compared to salbutamol only in the three subgroups included; however, the difference was statistically non-significant among overweight and normal subjects. In contrast, obese subjects showed significant improvement in spirometric results with p-values of 0.013, 0.002, and 0.035 for FEV_1_, FEV_1_% predicted, and FEV_1_/FVC, respectively. The changes in FEV_1_% of predicted and FEV_1_ (L) across the study groups were statistically significant, with p-values < 0.001 and 0.0014, respectively (Fig. 1). Also, the reversibility test was positive in 17% of the salbutamol group compared to 32% in the fluticasone/salmeterol group, and the difference was statistically significant (P = 0.018).

**Table 2:** Post-bronchodilator spirometry test results in salbutamol and Fluticasone/salmeterol groups

Variables	Salbutamol (*n* = 209)	Fluticasone/Salmeterol (*n* = 206)	P-value
FEV_1_ (L)			
Normal Overweight Obese	3.27 ± 1.71 3.04 ± 0.97 2.89 ± 1.12	3.85 ± 1.75 3.41 ± 1.22 3.37 ± 1.14	0.051 0.148 0.013*
FEV_1_% of predicted			
Normal Overweight Obese	95.28 ± 12.7 91.65 ± 14.7 89.93 ± 12.61	97.86 ± 11.9 95.32 ± 12.3 96.42 ± 11.34	0.221 0.116 0.002*
FEV_1_/FVC (%)			
Normal Overweight Obese	79.23 ± 17.2 78.64 ± 12.9 74.31 ± 15.3	83.32 ± 16.4 82.7 ± 14.9 79.6 ± 14.2	0.156 0.093 0.0354*
Positive Reversibility test *n* (%)	17 (8.1)	32 (15.6)	0.018*

FEV1: Forced expiratory volume in 1 s; FVC: Forced vital capacity

## Discussion

There is little concordance between guidelines regarding the bronchodilator reversibility test assessment standards [11, 12]. A few studies were designed to determine the optimum bronchodilator drug for the reversibility test [8, 9]. The present study takes precedence in comparing the effect of salbutamol versus fluticasone/salmeterol combination on the reversibility test and identifying the impact of body mass index on the spirometric outcomes. One of the harmful consequences of obesity is respiratory function impairment. However, the effect of BMI on spirometry was controversial in many clinical trials. The results of the present study showed lower spirometric values with an increase in BMI. In agreement with our results, many studies reported a consistent decrease in FEV_1_, FVC, and FEV_1_/FVC ratio with increased BMI [13, 14]. Contradictory to our results, a positive correlation of BMI with FVC values was reported amongst obese individuals in other studies, contributing to this correlation to the muscularity effect with age and height dependence [15, 16]. However, this effect was marked in group II and III obesity. This variability from our results can be attributed to dealing with obese subjects as a group without sub-categorization.

Overweight and obese subjects are more prone to misdiagnose asthma or chronic obstructive pulmonary disease (COPD) [17, 18]. The bronchodilator reversibility test is one of the lung laboratory’s most common diagnostic tools. Since the extent of response can be affected by several parameters, such as the bronchodilator type and dose, the administration technique, and the elapsed duration till the assessment of bronchodilator response, the method applied for assessing reversibility may be fundamental. However, there are no evidence-based standards regarding bronchodilator selection or the timing of post-bronchodilator response estimation [12, 19].

Short-acting- β2 agonists such as salbutamol or anticholinergics commonly use bronchodilators for reversibility testing in patients with suspected obstructive pulmonary disease. Long-acting β2 agonists have also been reported to exhibit a short-term action, and they can be applied for early reversibility tests. Using LABAs/ inhaled corticosteroids in early reversibility testing results in more remarkable improvement in FEV_1_ in patients with obstructive lung diseases [9]. In concordance with our results, which revealed that fluticasone and salmeterol combination was associated with more remarkable improvement in FEV1, FEV1% of predicted, and FEV1/FVC compared to salbutamol only in all subjects; however, these outcomes were only statistically significant for obese subjects.

The percentage of patients with a positive reversibility test was higher in the salmeterol/fluticasone group. The difference in the extent of the bronchodilation effect between the study groups may be explained by the combination of bronchodilators that can help bronchial dilation with different mechanisms and enhance the airway response. In addition, increasing evidence suggests that using LABA/inhaled corticosteroids in the same inhaler augments their effect on FEV_1_ rather than using separate inhalers [20], which is consistent with our findings.

However, in normal or overweight subjects, salbutamol inhalers can sufficiently achieve a bronchodilation response that is not significantly lower than that achieved by the LABA/corticosteroids combination in the reversibility test, in contrast to obese subjects, who need a combination of bronchodilator medications to increase the extent of bronchial airway dilation [21, 22] due to the mechanical effect of obesity on the respiratory airways [5]. It is worth noting that obesity was associated with lower responsiveness to different types of inhaled corticosteroids, including beclomethasone and budesonide, in obese asthmatic patients [23, 24], unlike fluticasone propionate, which showed significant efficacy in FEV1 improvement regardless of body weight [25].

Therefore, we suggest that salmeterol/fluticasone inhalation can be used in early reversibility testing instead of salbutamol to improve spirometry values and more reliable outcomes, especially in obese subjects.

Although the present study takes precedence in assessing the effect of fluticasone/salmeterol inhalation in early reversibility testing compared with the conventional approach of using salbutamol among obese participants, the study has several limitations.

Firstly, only drug choice was investigated in this study, limiting the measurement timing to 15 min after bronchodilator administration, which may be inadequate for accurately determining the maximal bronchodilation response. Secondly, the study was designed as a parallel-group study rather than a cross-over design, which may result in bias due to inter-individual variability and a lack of reproducibility due to the implications of many factors, such as the maneuver of inhaler use and measurement timing. Thirdly, the study lacks follow-up to ensure the sensitivity and accuracy of this modified approach to the bronchodilator reversibility test. Further extensive randomized studies with cross-over design and follow-up are warranted to validate the outcomes of this combination in obese patients.

## Conclusion

In conclusion, dual bronchodilator (fluticasone/salmeterol) use with variability in the mode of action resulted in a higher increase in FEV1, FEV1% of predicted, and FEV1/FVC ratio than the conventional test using salbutamol inhaler, with significant improvement in spirometry results in obese patients. This study raises the potential of using the fluticasone/salmeterol combination for reversibility testing instead of salbutamol alone, especially among obese subjects. Further studies are needed to assess the overall modified approach.

## Data Availability

The datasets used and/or analyzed during the current study are available from the corresponding author on reasonable request.
